# Associations Between Single and Multiple Vitamins and the Risk of Dental Caries: Results from a Cross-Sectional Study and Mendelian Randomisation Analysis

**DOI:** 10.3290/j.ohpd.c_2393

**Published:** 2025-12-12

**Authors:** Qingwen He, Hongyan Xu, Mali Liu, Qiuxia Yu, Mengyuan Lin

**Affiliations:** a Qingwen He Assistant Professor, Department of Public Health, Affiliated Women’s Hospital of Jiangnan University, Wuxi, 214002, China. Designed the study, collected data, contributed new methods, wrote the original manuscript, and approved the submitted version.; b Hongyan Xu Intern, Department of Public Health, Affiliated Women’s Hospital of Jiangnan University, Wuxi, 214002, China. Collected data, contributed new methods, and approved the submitted version.; c Mali Liu Dentist, Center of Stomatology, Affiliated Women’s Hospital of Jiangnan University, Wuxi, 214002, China. Designed the study, approved the submitted version.; d Qiuxia Yu Intern, Group Healthcare Department, Affiliated Women’s Hospital of Jiangnan University, Wuxi, 214002, China. Wrote and approved the submitted version.; e Mengyuan Lin Associate Professor, Center of Reproductive Medicine, Affiliated Women’s Hospital of Jiangnan University, Wuxi, 214002, China. Designed the study, contributed new methods, funding acquisition, wrote the final manuscript, and approved the submitted version.

**Keywords:** dental caries, inflammatory markers, Mendelian randomisation study, NHANES, vitamins

## Abstract

**Purpose:**

This study aimed to investigate the association between the multivitamins with dental caries.

**Materials and Methods:**

Our study investigated the association between eight vitamins (including vitamin A, B1, B2, B6, B12, C, K and E) with dental caries in the NHANES. The mixed effects of multiple vitamins on dental caries were assessed using weighted quantile sum (WQS) and Bayesian kernel machine regression (BKMR). Mediation analysis was performed to explore the role of inflammatory indicators in vitamin deficiency-induced dental caries. Mendelian randomisation (MR) analysis was utilised to determine the potential causal relationship between multivitamins and dental caries.

**Results:**

All the 5,145 individuals enrolled in our study, finally 1,715 were diagnosed with dental caries. The result of Model 3 after adjusting for all vitamins and covariates indicated that only vitamin B12 was negatively associated with dental caries in other three quartiles compared with the lowest quartile. Moreover, the dental caries risk decreased with increased concentration levels of multiple vitamins in the BKMR model. Vitamin B12 was confirmed as the main contributor to the association in WQS analysis. Mediation analysis indicated that four inflammatory indicators were the potential effects of vitamin B12 on dental caries. During MR analysis, a causality between vitamin B12 deficiency and dental caries was found.

**Conclusion:**

The cross-sectional study discovered a negative association between vitamin mixtures exposure and dental caries prevalence, with vitamin B12 as the main contributor. MR analysis also supported a causality between vitamin B12 deficiency and dental caries.

As the most prevalent oral disease affecting humanity globally of all ages, dental caries serves as the primary aetiology of tooth loss, causing a considerable oral health burden on humankind.^17,28^ Meanwhile, dental caries could also affect oral health and contribute to some other systemic diseases, such as cardiovascular diseases.^12,33,36^ Thus the prevention and treatment of dental caries is crucial for alleviating this global health risk.^34^

Vitamins are essential components of nutrition in health. In developing countries the deficiency of vitamins is a serious problem.^11,31^ Vitamins have long been considered a candidate for being an adjunct to dental caries,^3.^ but the relationship between vitamin levels and dental caries remains uncertain. Several recent studies have explored the relationship between vitamin levels and dental caries.^10,24^ In a cross-sectional study of 60 children, it was reported that children with dental caries had lower levels of salivary vitamin C and D compared to those without caries.^26^ However, no significant causal relationship of the levels of circulating vitamin C or D and dental caries was observed in a Mendelian randomisation study.^20^ Generally speaking, humans are usually exposed to a combination of multiple dietary vitamins, which may lead to synergy or antagonistic effects. However, at present most of the researches on nutritional epidemiology only evaluate the impact of individual vitamin intake on dental caries, rather than considering the co-intake of all vitamins.

Therefore, to assess the effects of eight vitamins intake (including vitamin A, vitamin B1, vitamin B2, vitamin B6, vitamin B12, vitamin C, vitamin K, vitamin E) on dental caries, we first conducted an observational study based on NHANES database. Whether inflammatory indicators have a potential role in mediating these associations was further examined. Furthermore, to estimate the evidence for a causal relationship between multivitamins and dental caries, we used publicly available genetic data with the framework of the MR analysis.

## MATERIALS AND METHODS

### Population

The data used in the study were publicly available from the NHANES database (https://www.cdc.gov/nchs/nhanes/index.htm). Informed consent was signed by all participants who participated in the data collection study. From three survey cycles in 2005–2006, 2007–2008 and 2009–2010, the NHANES data were selected for this cross-sectional study. Meanwhile, eight vitamins including vitamin A, vitamin B1, vitamin B2, vitamin B6, vitamin B12, vitamin C, vitamin K, vitamin E were quantified. This study only included participants who provided complete data following their response to the question about ‘Dental decay present’ and had detected vitamins levels. The participants with incomplete related data were excluded. Finally, totally 1,715 with dental caries and 3,430 controls were selected in the research. Figure S1 illustrated the sample selection process.

### Exposure and Outcome Variables

The vitamins’ concentration data used in the study were obtained from the dietary section of the 2005–2010 NHANES database. We chose eight vitamins on the first day’s total nutrient intake. It was a 24-h first meal recall interview collected in-person by the Mobile Examination Center (MEC). ‘Do you presently have dental caries? ’ data was collected by the Examination. Those who answered ‘yes’ were diagnosed with dental caries. The answered as ‘no’ participants were diagnosed as not having dental caries. The participants answered with ‘Could not assess’, and ‘Missing’ participants were excluded from the study. The process of obtaining the outcome variables can be found at https://www.cdc.gov/nchs/nhanes/index.htm

### Covariates

In our study, we incorporated clinically meaningful covariates, including age, ethnicity (Non-Hispanic Black, Mexican American, Non-Hispanic White, and Other), marital status (married/ living with partner, separated/ never/ refused, widowed/ divorced), education (college or above, high school or equivalent, less than high school), family poverty income ratio (PIR) (<1, ≥1), smoking status (nonsmoker, former smoker, current smoker), alcohol consumption (Yes, No), physical activity (inactive, moderate, vigorous or both moderate and vigorous), body mass index (BMI), personal history of diabetes, history of hypertension, and history of hyperlipidemia. The CRP (C-reactive protein), leukocyte, neutrophil, and lymphocyte counts (1,000 cells/uL) were analysed by the Beckman Coulter MAXM instrument.

### Statistical Analyses

In the study, based on the data of the NHANES sampling strategy, the 5-year sampling weights were constructed and incorporated into all analyses. The data were statistically analysed with the use of the R statistical software (version 4.4.0). The four inflammatory markers were Ln-transformed. Mean ± standard error (mean ± SE) was used to express the continuous variable, and a weighted t-test was conducted for comparison between the two groups. Frequency (composition ratio) n (%) was used to express categorical variables, and the chi-square test was conducted for comparison between two groups. Based on the distribution, eight single vitamin levels were divided into four quartiles (Q1, Q2, Q3, and Q4).

Weighted binary logistic regression analyses were conducted to estimate the association between single vitamin levels and dental caries. Based on the variable containing the median value, a test for trend was performed for each quartile. A test for linear trend was performed with the use of quartiles of the vitamin levels as a continuous variable by assigning the median values of the quartiles to the variable. Meanwhile, the restricted cubic spline (RCS) analysis^19^ was conducted to examine the nonlinear link between vitamin levels and dental caries. Model 1 was adjusted for age, BMI, smoking status, alcohol consumption, education, marital status, personal history of diabetes and PIR. Model 2 was adjusted for all eight vitamins with no covariate adjusted. Model 3 was adjusted for Model 1 plus other vitamins.

The correlations among eight vitamins were assessed by Spearman’s correlation analysis. WQS regression and BKMR analyses32 were performed on highly correlated vitamins. In our study, bootstrapping with 10,000 iterations was employed to construct WQS indexes in both negative and positive directions (R package (‘gWQS’)). Randomly, 40% of the data was divided into the training set, and the remaining 60% was divided into the validation set. When the WQS index showed statistical significance, we examined the corresponding weights to determine the relative contribution of each vitamin to dental caries. BKMR analysis was utilised to examine the comprehensive effects of co-exposure to vitamins on dental caries. Using the Markov Chain Monte Carlo algorithm, 10,000 iterations were performed for analysis (‘BKMR’packages).

‘Mediation’ package in R 4.4.0 was utilised to perform mediation analysis assessing the mediating effects of four inflammatory indicators on the associations of vitamin B12 and vitamin E with dental caries.

### Mendelian Randomisation Data Sources

A two-sample design and publicly available summary statistics from large-scale genome-wide association studies (GWAS) datasets were utilised in our MR analysis. For outcome data, we obtained genetic data from the GWAS Catalogue, which included 2,906 cases of European ancestry and 453,442 European ancestry controls. Table S4 provides detailed information on the GWAS summary level data of exposure and outcome analysed in this MR study.

### Selection of Genetic Instrumental Variants

Genetic instruments were extracted from relevant GWASs. We included 13, 17, 17, 8, 18, 9, 23 and 12 independent genetic variants in MR analysis for vitamin A, vitamin B1, vitamin B2, vitamin B6, vitamin B12, vitamin C, vitamin K and vitamin E, as shown in Table S4 and Table S5. Independent single-nucleotide polymorphisms (SNPs) for all vitamins were identified through GWAS at a significance threshold of P < 5 × 10-6. The next step involved determining the strength of each independent variable (IV) by calculating the F-statistics. Weak instruments were subsequently excluded. It was ensured in this MR study that each included IV has an F-statistic value >10. Strict criteria were applied, including LD clumping with r^2^ <0.001 and a window size of 10,000 kb. To ensure the specificity of SNPs, at the genome-wide significance level, we utilised the PhenoScanner tool (http://www.phenoscanner.medschl.cam.ac.uk/) to validate the exclusion of pleiotropic effects on other phenotypes. This process aimed to minimise the introduction of any confounding variables into the study findings.

### MR Analysis

In this two-sample MR analysis, genetic variants were utilised as IVs to determine if eight vitamins have a causative effect on dental caries. The main approach employed in this study was the inverse-variance weighted (IVW) method.^7^ Weighted Median, MR-Egger, Maximum Likelihood, Simple Median, and Penalised Weighted Median were employed to ensure the stability and reliability of the data.^4^

Besides the primary analyses, sensitivity analyses were conducted to ensure the robustness of the results. Using the MR-PRESSO test and the MR-Egger intercept, we assessed potential horizontal pleiotropic effects on the instrumental variables (IVs). The MR-PRESSO test was adjusted for SNP outliers, with NbDistribution set to 10,000 in our study. Moreover, Cochran’s Q test and funnel plots were used to examine the SNP heterogeneity.^6^

Additionally, we conducted a ‘leave-one-out’ sensitivity analysis to investigate whether the causal association was influenced by any individual SNP and to ensure the robustness of the results. We conducted statistical analyses of the odds ratio (OR) with 95% confidence interval (CI) for this outcome using the R version 4.4.0, with the R packages ‘Two-Sample MR’, ‘Mendelian Randomisation’, and ‘MR-PRESSO’.

## RESULTS

### Population Characteristics and Clinical Data

**Fig 1a to e:**
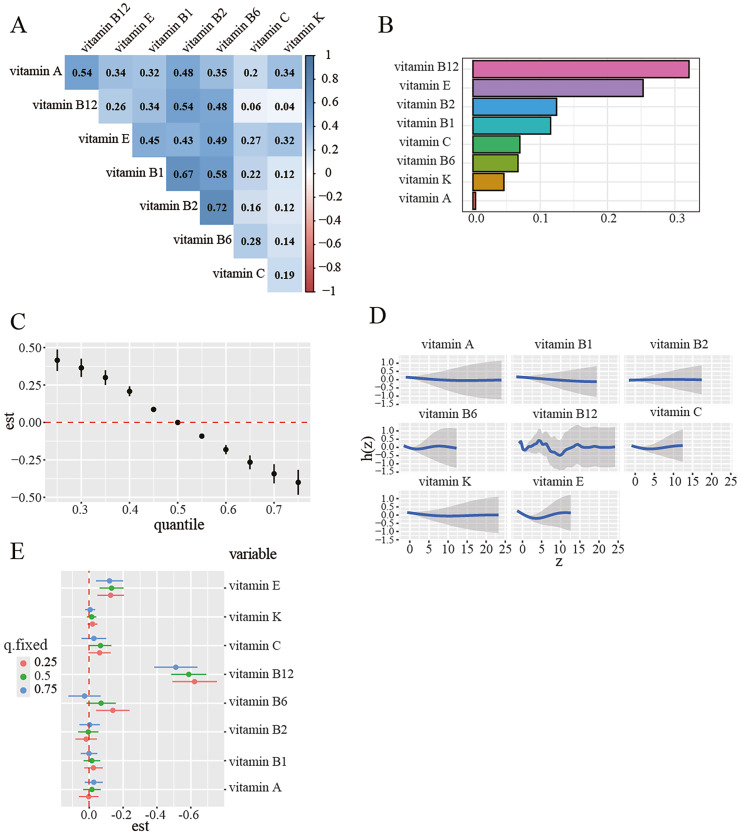
The mixed effects of 8 vitamins on dental caries were assessed using weighted quantile sum (WQS) and Bayesian kernel machine regression (BKMR) model. Models were adjusted for age, body mass index, smoking status, alcohol consumption, education, marital status, personal history of diabetes, family poverty income ratio and all vitamins. (a) Spearman’s correlation analysis among the 8 single vitamins. all P <0.001. (b)The WQS model weights of vitamins on dental caries in a negative direction. (c) Combined effects of an eight-vitamin mixture on dental caries were estimated by the BKMR models. (d) Univariate exposure-response function between each vitamin and the prevalence of dental caries when the other vitamins were fixed at the 50th percentiles. (e) Associations of eight single vitamins with dental caries were estimated by BKMR, when all other vitamins were held at their corresponding 25th, 50th or 75th percentile, respectively.

The baseline characteristics of the subjects are presented in Table 1. A total of 5,145 subjects from NHANES 2005-2010 were included in this study, which included 1,715 dental caries and 3,430 controls. Figure S1 illustrates the sample selection process. Furthermore, compared to the controls, the dental caries group demonstrated a higher tendency to suffer from obesity, a lower education level, and lower family income, as well as higher values of CRP, leukocytes, neutrophils, and lymphocytes. In Spearman’s correlation analysis, a positive correlation was revealed among eight vitamins in Figure 1a. Vitamin B2 was found to significantly correlate with vitamin B1, vitamin B6 and vitamin B12 (r = 0.67;0.72 and 0.54, all P <0.001, respectively), vitamin A and vitamin B12 were correlated as well (r = 0.54, P <0.001).

**Table 1:** Characteristics of participants

Characteristics	Overall (n = 5,145)	Control (n = 3430)	Dental caries (n = 1,715)	P value
**Age (mean (SE))**	44.82 (16.10)	45.78 (16.19)	42.64 (15.67)	<0.001
**Gender,n (%)**				0.534
Male	2741 (55.8)	1788 (55.5)	953 (56.7)	
Female	2404 (44.2)	1642 (44.5)	762 (43.3)	
**Education,n(%)**				<0.001
Less than high school	597 (6.2)	341 (5.1)	256 (8.7)	
High school or equivalent	831 (12.0)	479 (9.8)	352 (16.9)	
College or above	3715 (81.8)	2610 (85.0)	1105 (74.4)	
**Marital status,n (%)**				<0.001
Widowed/Divorced	916 (14.7)	601 (14.1)	315 (16.0)	
Separated/Never/Refused	1116 (21.3)	689 (19.4)	427 (25.6)	
marriageMarried/Living with partner	3113 (64.0)	2140 (66.5)	973 (58.3)	
**BMI (kg/m** ^2^ **)(mean (SE))**	28.55 (6.32)	28.32 (6.04)	29.09 (6.88)	0.007
**Smoking status,n (%)**				<0.001
Non-smoker	2773 (53.5)	1965 (57.7)	808 (44.0)	
Former smoker	1279 (24.2)	972 (26.1)	357 (20.1)	
Current smoker	1093 (22.3)	543 (16.2)	550 (35.9)	
**Alcohol consumption, n (%)**				0.016
YES	550 (12.9)	393 (13.9)	157 (10.5)	
NO	4595 (87.1)	3037 (86.1)	1558 (89.5)	
**Personal history of diabetes, n (%)**				0.026
YES	589 (7.9)	383 (7.1)	206 (9.6)	
NO	4556 (92.1)	3047 (92.9)	1509 (90.4)	
**Hypertension history, n (%)**				0.615
YES	1691 (28.3)	1140 (28.1)	551 (28.9)	
NO	3454 (71.7)	2290 (71.9)	1164 (71.1)	
**Hyperlipidemia history, n (%)**				0.249
YES	1560 (41.7)	1131 (42.2)	429 (40.1)	
NO	2017 (58.3)	1429 (57.8)	588 (59.9)	
**PIR, n (%)**				<0.001
<1	1287 (18.1)	709 (9.7)	578 (21.6)	
≥1	3858 (81.9)	2721 (90.3)	1137 (78.4)	
**Physical activity，n (%)**				0.511
Inactive	1936 (31.2)	1227 (30.8)	659 (32.0)	
Moderate	1616 (33.4)	1121 (34.3)	495 (31.5)	
Vigorous	359 (7.5)	236 (7.4)	123 (7.7)	
Both moderate and vigorous	1234 (27.9)	796 (27.5)	438 (28.8)	
**Vitamin A, mcg (mean (SE))**	720.56 (642.89)	774.25 (581.80)	599.68 (748.96)	<0.001
**Vitamin B1, mg (mean (SE))**	1.93 (1.28)	2.06 (1.39)	1.63 (0.92)	<0.001
**Vitamin B2, mg (mean (SE))**	2.62 (1.53)	2.80 (1.58)	2.22 (1.33)	<0.001
**Vitamin B6, mg (mean (SE))**	2.41 (1.50)	2.58 (1.53)	2.01 (1.33)	<0.001
**Vitamin B12, mg (mean (SE))**	7.32 (7.07)	8.04 (6.68)	5.72 (7.66)	<0.001
**Vitamin C, mg (mean (SE))**	95.85 (101.22)	100.79 (95.98)	84.74 (111.37)	0.001
**Vitamin K, mcg (mean (SE))**	106.77 (154.29)	113.90 (158.41)	90.72 (143.32)	<0.001
**Vitamin E, mg (mean (SE))**	8.38 (6.00)	9.09 (6.23)	6.79 (5.10)	<0.001
**ln (CRP) (mean (SE))**	–1.77 (1.26)	–1.83 (1.26)	–1.63 (1.26)	<0.001
**ln (leukocyte) (mean (SE))**	1.94 (0.28)	1.92 (0.28)	1.99 (0.29)	<0.001
**ln (neutrophil) (mean (SE))**	1.38 (0.38)	1.36 (0.37)	1.43 (0.39)	0.001
**ln (lymphocyte) (mean (SE))**	0.72 (0.32)	0.69 (0.32)	0.77 (0.31)	<0.001
Abbreviations: SE, standard error; BMI, body mass index; PIR, family poverty income ratio; CRP, C-reactive protein.

#### Vitamin exposures and dental caries in the weighted binary logistic regression model

Results of weighted binary logistic regression analyses among single vitamin intake in relation to dental caries are displayed in Table 2. In the crude model without controlling for any covariates, eight dietary vitamins intake were significantly negatively correlated with dental caries in all quartiles (all P for trend <0.05). In Model 1, except for vitamin C, other vitamins were significantly negatively correlated with dental caries in the second, third and highest quartiles than the lowest quartile (all p for trend<0.05). In Model 2, we found that higher intake levels of vitamin B12 (Q2, OR = 0.40, 95%CI: 0.29-0.55; Q3, OR = 0.23, 95%CI: 0.16-0.33; Q4, OR = 0.27, 95%CI: 0.18-0.40) and vitamin E (Q3, OR = 0.62, 95%CI: 0.50-0.78; Q4, OR = 0.62, 95%CI: 0.47-0.80) were related to a decreased risk of developing dental caries compared to Q1 (all p for trend<0.05). In Model 3, adjusted for all covariates and other vitamins, we found that higher intake levels of dietary vitamin B12 (Q2, OR = 0.74, 95%CI: 0.62-0.88; Q3, OR = 0.49, 95%CI: 0.39-0.60; Q4, OR = 0.52, 95%CI: 0.40-0.66) and vitamin E (Q3, OR = 0.71, 95%CI: 0.58-0.87; Q4, OR = 0.71, 95%CI: 0.56-0.90) were related to a decreased risk of developing dental caries compared to lower intake levels (all p for trend<0.05). Other vitamins showed no significant difference between the two groups in Model 2 and Model 3 (all P for trend > 0.05).

**Table 2:** The association between concentrations of eight vitamins and the odds of dental caries

Variables	Crude model	Model 1	Model 2	Model 3
Crude OR (95% CI)	P value	Adjusted OR (95% CI)	P value	Adjusted OR (95% CI)	P value	Adjusted OR (95% CI)	P value
Vitamin A (median[*range*])								
Q1 (199[<340])	Reference		Reference		Reference		Reference	
Q2 (464[340, 595])	0.58 (0.49, 0.69)	7.12E-07	0.65 (0.55, 0.78)	9.65E-05	0.97 (0.76, 1.22)	0.733	0.98 (0.82, 1.17)	0.853
Q3 (733[595, 912])	0.43 (0.35, 0.53)	2.62E-09	0.54 (0.44, 0.66)	6.65E-06	0.99 (0.72, 1.35)	0.933	1.12 (0.91, 1.37)	0.299
Q4 (1238[>-0.55])	0.31 (0.24, 0.40)	3.12E-10	0.40 (0.31, 0.53)	1.57E-06	0.86 (0.59, 1.24)	0.355	0.98 (0.77, 1.24)	0.848
*p* for trend	6.58E-10		2.56E-06		0.126		0.814	
Vitamin B1 (median[range])								
Q1 (0.9[<1.193])	Reference		Reference		Reference		Reference	
Q2 (1.43[1.193, 1.687])	0.63 (0.53, 0.75)	4.74E-06	0.71 (0.59, 0.86)	1.37E-03	1.08 (0.86, 1.37)	0.454	1.05 (0.88, 1.26)	0.580
Q3 (1.96[1.687, 2.349])	0.47 (0.41, 0.55)	1.45E-10	0.56 (0.47, 0.66)	8.68E-07	1.10 (0.87, 1.39)	0.363	1.01 (0.82, 1.26)	0.875
Q4 (2.94[>2.349])	0.33 (0.25, 0.42)	6.62E-10	0.37 (0.28, 0.49)	8.05E-07	0.97 (0.64, 1.47)	0.862	1.05 (0.81, 1.37)	0.682
*p* for trend	1.15E-10		1.61E-07		0.573		0.676	
Vitamin B2 (median[range])								
Q1 (1.205[<1.666])	Reference		Reference		Reference		Reference	
Q2 (2.013[1.666, 2.372])	0.55 (0.44, 0.69)	7.01E-06	0.60 (0.45, 0.78)	8.66E-04	1.04 (0.76, 0.99)	0.78	0.99 (0.82, 1.20)	0.934
Q3 (2.742[2.372, 3.278])	0.31 (0.25, 0.39)	1.88E-11	0.35 (0.27, 0.44)	8.27E-08	0.90 (0.64, 1.25)	0.455	0.78 (0.61, 1.00)	0.054
Q4 (4.022[>3.278]])	0.29 (0.23, 0.36)	5.47E-12	0.31 (0.24, 0.40)	3.67E-08	1.10 (0.76, 1.59)	0.568	0.96 (0.70, 1.30)	0.778
*p* for trend	3.80E-12		6.35E-09		0.843		0.350	
Vitamin B6 (median[range])								
Q1 (1.031[<1.485])	Reference		Reference		Reference		Reference	
Q2 (1.786[1.485, 2.112])	0.48 (0.37, 0.61)	1.19E-06	0.53 (0.42, 0.67)	2.34E-05	0.79 (0.59, 1.05)	0.094	0.8 (0.67, 0.96)	0.014
Q3 (2.46[2.112, 2.949])	0.33 (0.26, 0.41)	1.10E-10	0.36 (0.28, 0.45)	6.38E-08	0.81 (0.59, 1.11)	0.153	0.88 (0.71, 1.09)	0.227
Q4 (3.8[>2.949])	0.27 (0.21, 0.35)	3.90E-11	0.30 (0.22, 0.40)	1.75E-07	0.90 (0.60, 1.36)	0.577	0.88 (0.67, 1.14)	0.322
*p* for trend	1.29E-11		4.55E-08		0.695		0.576	
Vitamin B12 (median[range])								
Q1 (2.11[<3.69])	Reference		Reference		Reference		Reference	
Q2 (5.09[3.69, 6.1])	0.33 (0.27, 0.42)	1.65E-10	0.36 (0.28, 0.46)	1.08E-07	0.40 (0.29, 0.55)	2.24E-04	0.74 (0.62, 0.88)	0.001
Q3 (7.15[6.1, 8.73])	0.19 (0.14, 0.24)	4.66E-14	0.21 (0.16, 0.27)	6.43E-10	0.23 (0.16, 0.33)	2.62E-05	0.49 (0.39, 0.60)	<0.001
Q4 (11.73[>8.73])	0.20 (0.16, 0.25)	1.20E-15	0.20 (0.16, 0.26)	1.78E-10	0.27 (0.18, 0.40)	9.73E-05	0.52 (0.40, 0.66)	<0.001
*p* for trend	<2e-16		2.56E-11		3.14E-08		3.24E-06	
Vitamin C (median[range])								
Q1 (12.8[<25.9])	Reference		Reference		Reference		Reference	
Q2 (42.1[25.9, 63.7])	0.66 (0.49, 0.87)	5.00E-03	0.82 (0.62, 1.09)	0.166	0.89 (0.64, 1.23)	0.418	1.06 (0.89, 1.27)	0.515
Q3 (94[63.7, 133.6])	0.54 (0.39, 0.73)	3.43E-04	0.68 (0.50, 0.93)	0.019	0.99 (0.68, 1.47)	0.063	0.97 (0.81, 1.17)	0.766
Q4 (202.8[>133.6])	0.46 (0.35, 0.61)	6.02E-06	0.60 (0.45, 0.80)	0.001	1.06 (0.72, 1.55)	0.018	0.88 (0.73, 1.07)	0.207
*p* for trend	6.64E-06		7.06E-04		0.008		0.107	
Vitamin K (median[range])								
Q1 (24.4[<37.1])	Reference		Reference		Reference		Reference	
Q2 (48.9[37.1, 62])	0.70 (0.58, 0.85)	5.31E-04	0.78 (0.63, 0.97)	0.028	0.98 (0.78, 1.24)	0.857	0.95 (0.79, 1.13)	0.551
Q3 (80.8[62, 113.6])	0.56 (0.46, 0.69)	3.95E-06	0.66 (0.53, 0.82)	6.85E-04	1.03 (0.77, 1.38)	0.827	0.90 (0.74, 1.09)	0.267
Q4 (192.7[>113.6])	0.48 (0.38, 0.60)	4.37E-07	0.59 (0.47, 0.74)	1.41E-04	0.98 (0.73, 1.31)	0.87	0.88 (0.71, 1.09)	0.252
*p* for trend	4.85E-06		4.30E-04		0.576		0.670	
Vitamin E (median[range])								
Q1 (3.145[<4.52])	Reference		Reference		Reference		Reference	
Q2 (5.63[4.52, 6.9])	0.66 (0.55, 0.81)	1.59E-04	0.76 (0.61, 0.94)	0.013	0.89 (0.70, 1.12)	0.268	0.86 (0.72, 1.02)	0.090
Q3 (8.37[6.9, 10.58])	0.38 (0.33, 0.45)	5.18E-13	0.42 (0.36, 0.49)	7.75E-10	0.62 (0.50, 0.78)	1.78E-03	0.71 (0.58, 0.87)	0.001
Q4 (14.02[>10.58])	0.31 (0.25, 0.38)	2.03E-11	0.37 (0.29, 0.47)	8.63E-08	0.62 (0.47, 0.80)	3.50E-03	0.71 (0.56, 0.90)	0.004
*p* for trend	4.09E-12		1.61E-08		1.92E-04		9.60E-04	
Abbreviations: OR, odds ratio; CI, confidence interval; Crude, no covariate was adjusted. Model 1 adjusted for age, BMI, smoking status, alcohol consumption, education, marital status, personal history of diabetes, PIR. Model 2 adjusted for all eghit vitamins with no covariate was adjusted. Model 3 adjusted for Model 1 plus other vitamins. Test for trend based on variable containing median value for each quartiles.

**Fig 2a to h:**
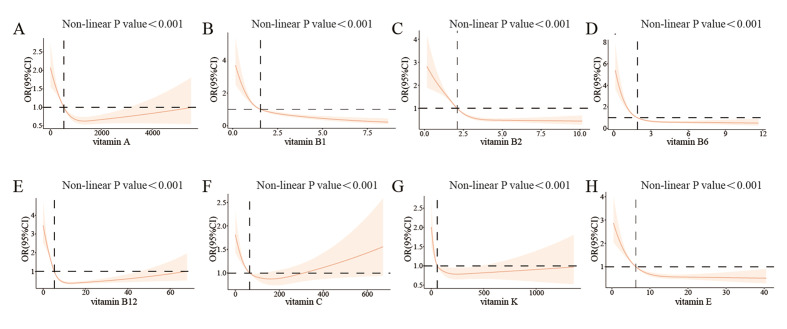
Restricted cubic spline plot of the association between vitamins and dental caries. (a) vitamin A; (b) vitamin B1; (c) vitamin B2; (d) vitamin B6; (e) vitamin B12; (f) vitamin C; (g) vitamin K; (h) vitamin E.

RCS was employed to reveal the dose–response relationship between eight dietary vitamins and dental caries in Model 1. As depicted in Figures 2a to 2h, the RCS curve displayed a non-linear and negative association of vitamin A, B1, B2, B6, B12, C, K, E with dental caries (non-linear P value <0.001).

#### Multivitamins exposure and dental caries risk in the WQS model

We utilised the WQS model to estimate the association between the combined effects of the eight vitamins with dental caries. As shown in Table S1, the WQS index showed a significant and negative association with the odds of dental caries (OR: 0.577; 95% CI: 0.52, 0.64;p < 0.001) in Model 3 when the WQS index was set to the direction of the negative effect. When the WQS index was adjusted for the positive effect, there was no statistically significant association observed between the WQS index and the odds of dental caries. As shown in Figure 1b, vitamin B12 received the highest weight of 0.321, followed by vitamin E for dental caries in Model 3.

#### Multivitamins exposure and dental caries risk in the BKMR model

In the BKMR model, the overall impact of eight vitamins on dental caries showed a downward trend, and an increase in the total level of vitamins mixture was associated with a decreased risk of dental caries (Fig 1c). Table S2 provided a summary of posterior inclusion probabilities (PIPs) in the BKMR model, with vitamin B12 intake exhibiting the highest PIP (0.998) for risk of dental caries. The univariate exposure-response function of all eight vitamins and dental caries is shown in Figure 1d. Furthermore, Figure 1e showed the negative effect of vitamin B12 level on dental caries prevalence when controlling for the 25th, 50th, and 75th percentiles of other vitamins.

#### Inflammation mediated the association between vitamins and dental caries

**Fig 3:**
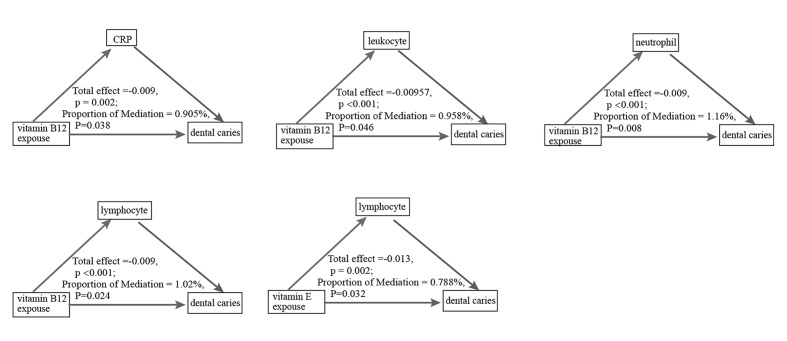
Mediating effect of inflammatory markers on vitamins and dental caries risk. Adjusted for age, body mass index, smoking status, alcohol consumption, education, marital status, personal history of diabetes and family poverty income ratio.

As shown in Figure 3 and Table S3, CRP, leukocyte, neutrophil and lymphocyte were significantly mediated the association between vitamin B12 and dental caries risk (proportion mediated (PM), 0.91%, P = 0.038; PM, 0.958%, P = 0.046; PM, 1.16%, P = 0.008; PM, 1.02%, P = 0.024).

Furthermore, only lymphocytes significantly mediated the association between vitamin E and dental caries risk (PM, 0.79%, P = 0.032). Additionally, we also assessed the mediating roles of vitamin E with other inflammatory indicators (Table S3).

#### MR of vitamins and dental caries

**Fig 4:**
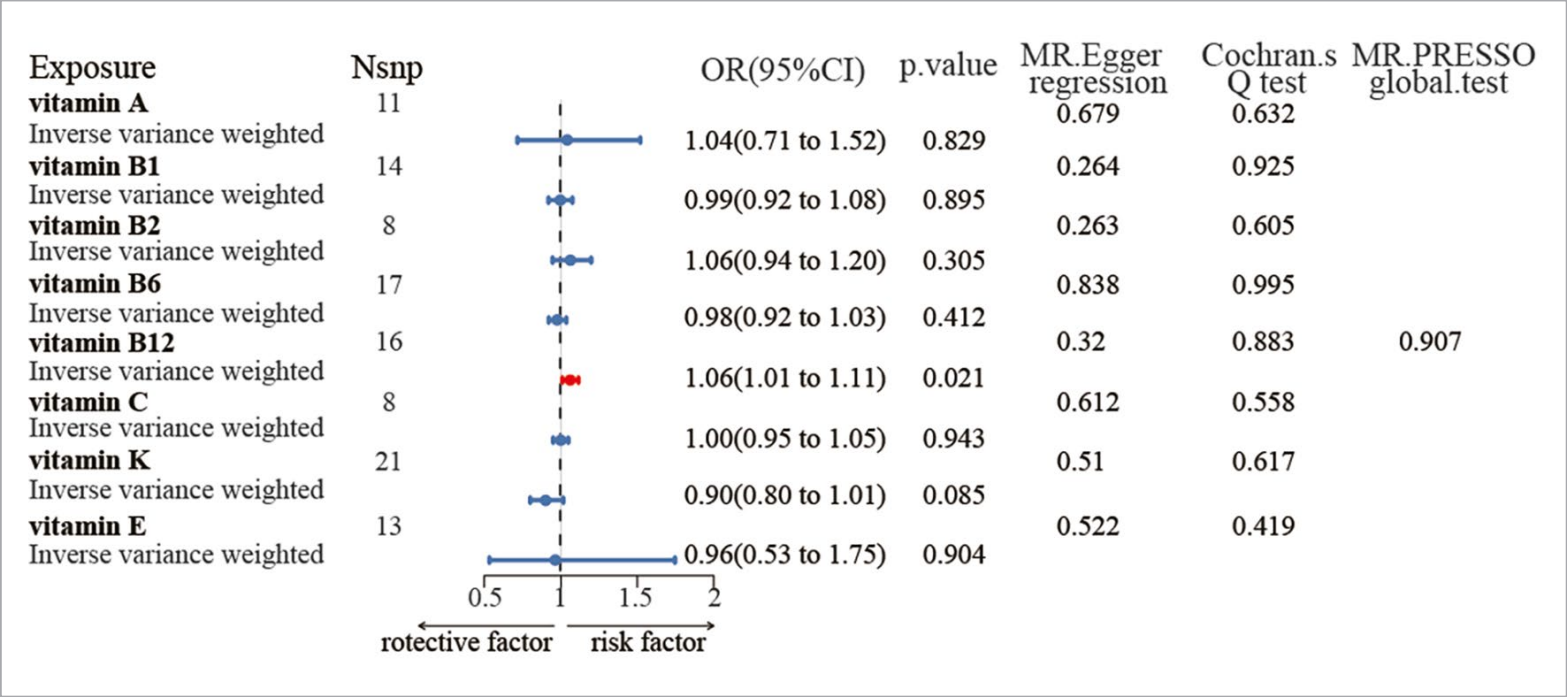

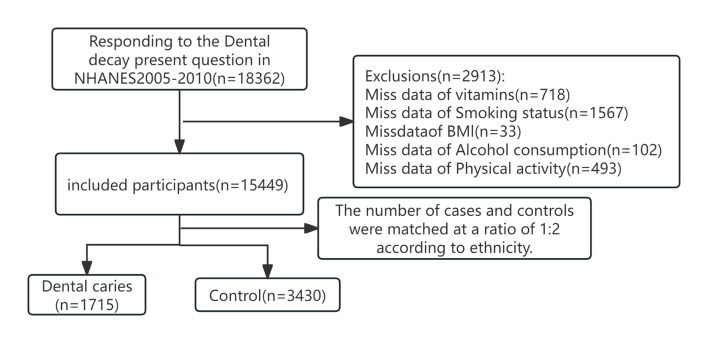

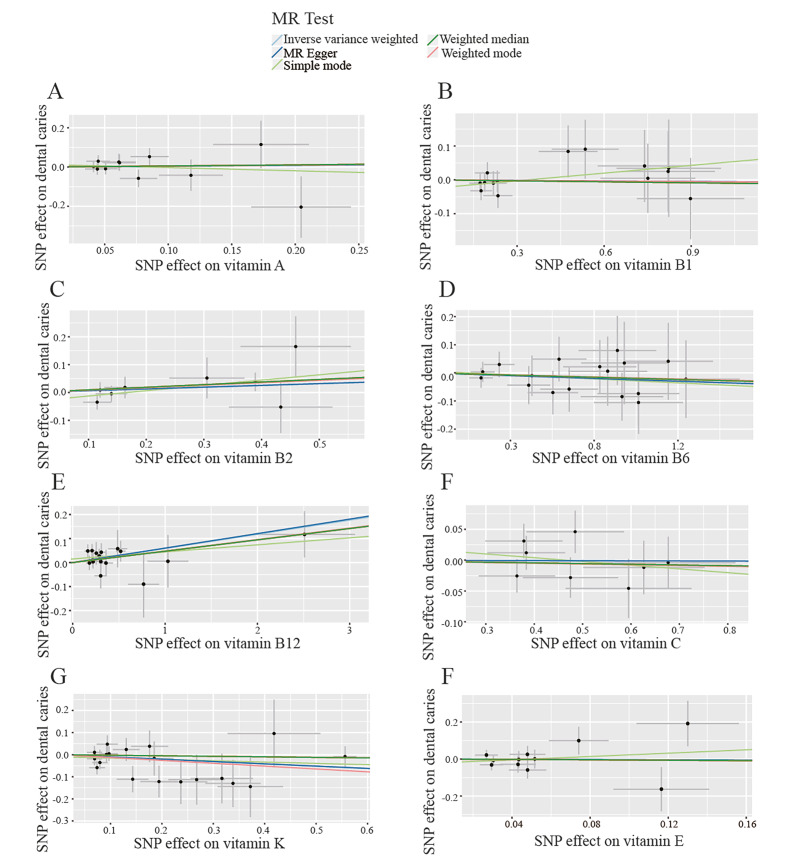

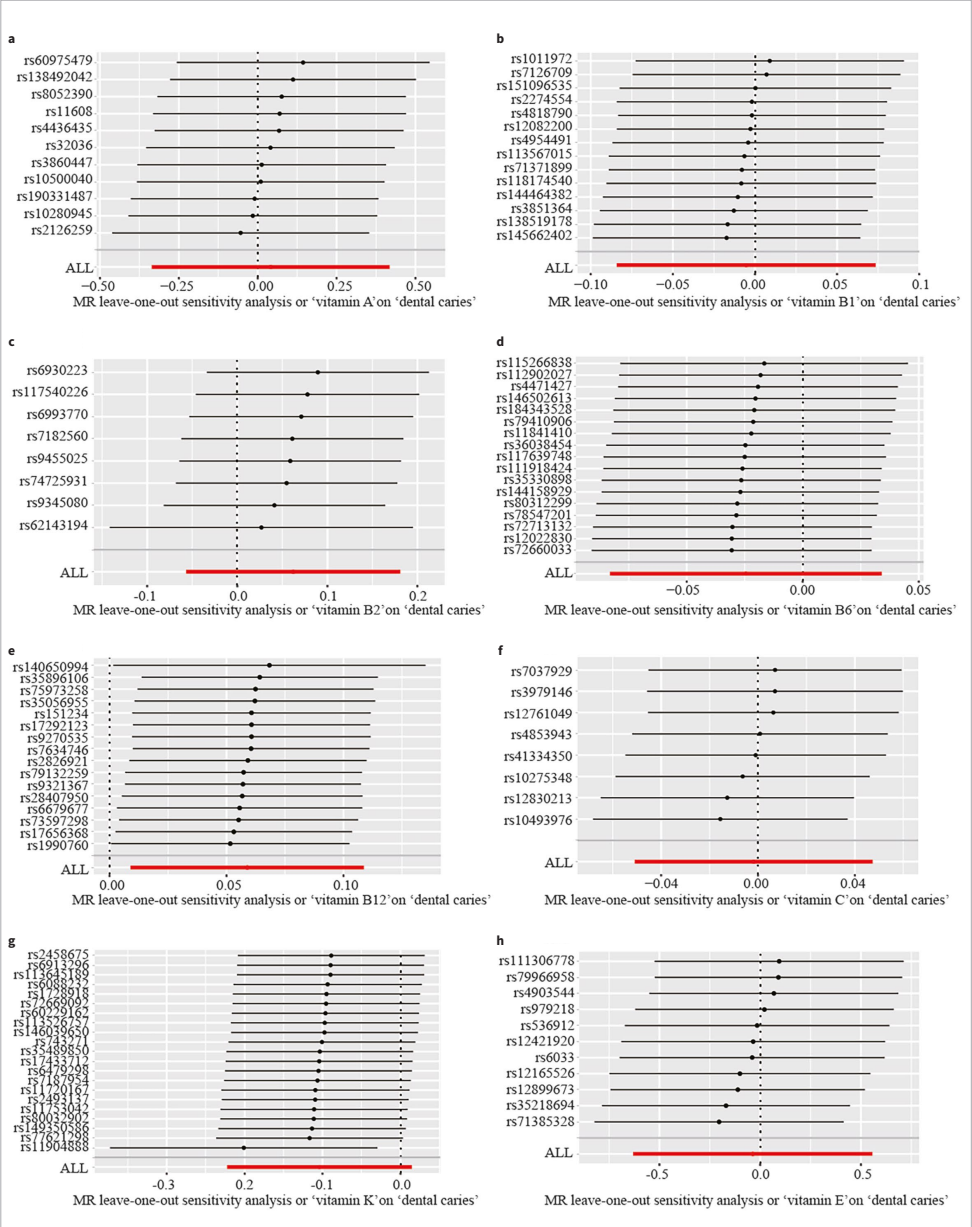

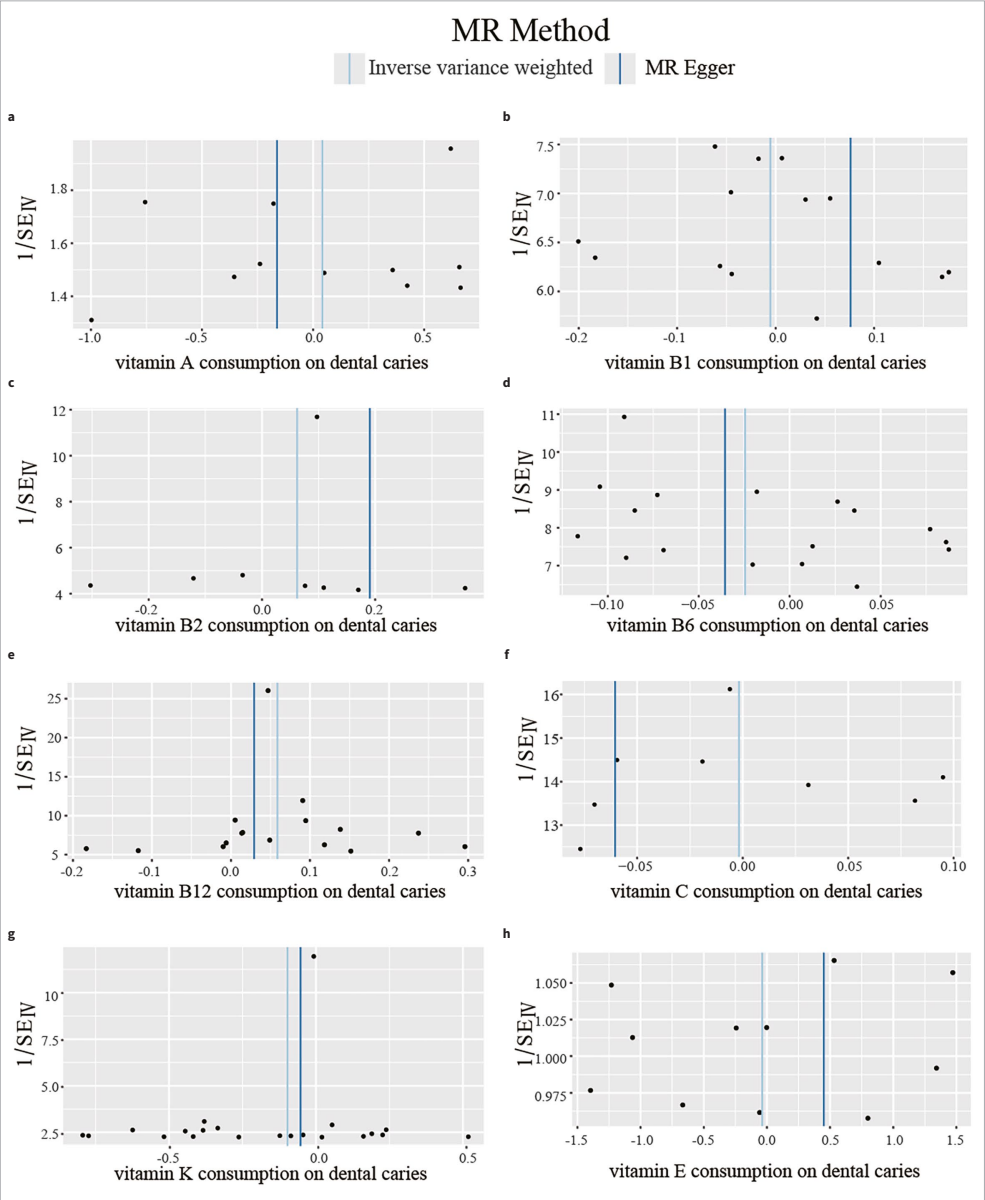
MR results for the relevance of vitamins to dental caries. OR, odds ratio; CI, confidence interval; Nsnp, number of SNPs.

As a significant correlation between vitamins and dental caries was observed in the above analysis, we further conducted MR analysis to deduce the causal effects of eight vitamins on dental caries. As shown in Figure 4, the MR analysis of the IVW method revealed that there was a positive causal relationship between vitamin B12 deficiency and dental caries risk (OR = 1.061, 95% CI:1.009–1.115). The analysis results of the other five methods (MR-Egger, Maximum Likelihood, Simple Median, Weighted Median and Penalised Weighted Median) were consistent in the direction of the effect with the IVW method (Table S5).

The P values of the MR-Egger intercept and MR-PRESSO global test indicated no significant evidence of directional pleiotropy (0.883 and 0.907). Additionally, the Cochran’s Q test did not reveal any significant heterogeneity (P = 0.32) (Fig 4). The scatter plot of the above eight vitamins was depicted in Figure S2. Furthermore, we performed a ‘leave-one-out’ sensitivity analysis to identify potentially influential SNPs (Fig S3). The estimates were not biased by a single SNP of vitamin B12. Most funnel plots were symmetrical (Fig S4). No significant associations were found between the other vitamins and dental caries, except for vitamin B12 (Fig 4).

## DISCUSSION

We explored the roles of dietary vitamin intake in dental caries in this cross-sectional study and Mendelian randomisation analysis. Our findings indicated that vitamin B12 was negatively associated with dental caries. Therefore, the findings of this study have important implications for adverse dental caries. In WQS analysis, the dental caries risk decreased with increased concentration levels of the mixtures of multiple vitamins in the BKMR model, and vitamin B12 (32.1%) was confirmed as the main contributor to the association. Mediation analysis indicated that four inflammatory indicators (CRP, leukocyte, neutrophil, lymphocyte) mediated the association between vitamin B12 and dental caries. The MR analysis also indicated a causal association of vitamin B12 deficiency and dental caries risk, while the MR effect size for vitamin B12 is very small (OR≈1.06), and mediation effects are almost <2%.

Vitamin B12 is one of the essential elements that the body cannot produce. Crowds mainly obtained vitamin B12 from animal products, such as eggs, fish, meat and milk. Vitamin B12 is important during a child’s early growth, for it may affect the development and function of the brain, memory, metabolism, attention and oral hygiene.^24,32^ Hugar et al^21^ reported that dental caries prevalence and associated gingival problems were increased in children of systemic vitamin B12 deficiency, which was consistent with our study results. Meanwhile, Chapple et al^10^ also found that vitamin B12 deficiencies may be related to the onset and progression of dental caries. We found that inflammatory indicators mediated the association of vitamin B12 deficiency with dental caries risk. In caries, the process of fermentation could cause acid production or the generation of biofilm components, such as glucans. It was found by Pontes et al that angular cheilitis, glossitis, recurrent oral ulcer, oral candidiasis, diffuse erythematous mucositis, and pale oral mucosa occurred in a case of vitamin B12 deficiency.^27^ Thus, we considered that vitamin B12 deficiency may cause an imbalance of oral hygiene, which leads to dental caries. At the same time, the vitamin B12 deficiency could cause megaloblastic anaemia.^9,29^ In addition, a significant association between anaemia and dental caries has been shown by numerous studies.^1,13,30^ So we thought vitamin B12 deficiency may also cause dental caries due to megaloblastic anaemia. Besides, our MR analysis indicated a causal association of vitamin B12 deficiency with dental caries risk. However, the OR of MR-derived is 1.06 and mediation effects are almost <2%, indicating a very small effect per genetically predicted unit change and against overinterpreting the MR results as strong causal evidence.

Vitamin E was known to serve in an antioxidant capacity, widely used in the prevention and treatment of chronic inflammatory diseases.^35^ Although many studies have shown an association between antioxidants, periodontitis and dental caries, the conclusions of relevant studies are contradictory.^14,21,31^ In our observational study, dietary vitamin E intake was associated with decreased odds of dental caries. However, no association between genetically predicted vitamin E and dental caries risk was observed in our MR analysis, which was consistent with Gao et al’s^18^ study results. One explanation could be that this study comes from cross-sectional studies, which tend to be limited by small sample sizes, confounding factors, reverse causality and other biases, leading to spurious associations.

Many other vitamins are reported to maintain oral hygiene and health. Some research has evaluated that vitamin D deficiency can lead to dental caries in primary and permanent teeth among children.^22^ Improving the status of vitamin D could be a beneficial strategy for prevention against dental caries among adolescents.^5,8,15^ Vitamin D deficiency during pregnancy may cause intrauterine enamel hypoplasia, and throughout childhood is accompanied by insufficient activity of antibacterial peptides, decreased saliva secretion, and a low level of calcium in saliva. Vitamin D deficiency would increase the risk of caries in the primary and/or permanent dentition. The relationship between vitamin D deficiency and dental caries is evident enough for vitamin D deficiency to be considered a risk factor for dental caries. Thus, vitamin D was excluded from our study of vitamins on the influence of dental caries.

As another essential nutrient, vitamin C has been found necessary to maintain many aspects of human health. Such as against scurvy, contributing to immunity and cardiovascular health, cancer and chronic disease prevention, many of vitamin C’s functions were attributed to its antioxidant effects.^23,25^ Zehdi et al^16^ reported that vitamin C caused a passive effect on biofilm formation and S. mutans growth, which were the key steps of dental caries. Although some scholars have observed high levels of oxidative stress and proteomic inflammatory markers in the saliva and serum of subjects with dental caries.^2,24^ A Mendelian randomisation study found no significant association between circulating antioxidants (vitamin C, zinc, ascorbate, copper, selenium, β-carotene, lycopene and retinol) and dental caries risk.^18^ Meanwhile, our results of the cross-sectional study and Mendelian randomisation analysis did not find the relationship between vitamin C and dental caries.

Our study explored the combined effect between vitamins and dental caries risk, and we hope our results could provide some references for the exploration of vitamin supplements to prevent and control dental caries. Based on the NHANES database using multi-stage complex sampling, the study sample size was large and had a good representation of the United States population. However, there are still some limitations in the current study. While we adjusted for as many covariates as possible to reduce the bias caused by confounding factors. Data on dietary vitamins intake information were collected through the NHANES 24-hour dietary recalls with questionnaires, which makes it hard to avoid the recall bias, while we only included the first day’s records to minimise the recall bias. In the study, we used systemic biomarkers to explore the mediation analysis. However, oral hygiene imbalance refers to behaviours and microbial plaque accumulation factors. Inflammation is a physiological/pathological immune response that may result from poor oral hygiene, but is not equivalent to it. These are systemic biomarkers and do not directly reflect oral hygiene status. The used GWAS and NHANES data are generated from individuals of Western descent, which may exhibit racial and regional heterogeneity that may affect the generalizability of the results. Thus, it is uncertain that our findings are applicable to other populations.

## CONCLUSIONS

This study demonstrates that a negative causal association between vitamin B12 and dental caries, and four inflammatory indicators mediated the association of vitamin B12 with dental caries risk. Considering the potential effects of vitamin B12 on dental caries, the clinical significance of four inflammatory indicators was minimal. Our results suggested a vitamin B12 supplement as potentially significant for the prevention and control of dental caries. More prospective studies should be conducted to confirm the relationship between vitamin B12 and the prevalence of dental caries in the future.

### Acknowledgements

#### Data availability

The present study is based on freely available summary statistics from GWAS and NHANES.

#### Declaration of competing interest

The authors declare that they have no conflict-of-interests.

#### Funding declaration

This research was supported by the project of the Science Foundation of Health Commission of Wuxi (No. Q202403).

## Appendix

**Fig A1:** Flow chart for participants’ recruitment of this study, NHANES 2005–2010.BMI, body mass index.

**Fig A2:** Scatter plots of the MR estimates for the significant causality of eight vitamins and dental caries.

**Fig A3:** Leave-one-out analyses for the causal estimates of eight vitamins on dental caries.

**Fig A4:** Funnel plots from eight vitamins on dental caries.

**Table A1:** The joint effect of vitamins mixtures on the prevalence of dental caries in WQS model.

Intercept	Estimate	Std.Erro	z value	P	OR(95%CI)
wqs	-0.549	0.052	-10.5	0	0.577 (0.521,0.64)
Age	-0.014	0.003	-4.92	0	0.986 (0.98,0.99)
BMI	0.011	0.006	1.76	0.078	1.011 (0.99,1.02)
Smoking status(Formersmoker)	-0.691	0.122	-5.65	0	0.501 (0.394,0.637)
Smoking status (Nonsmoker)	-0.859	0.102	-8.46	0	0.424 (0.35,0.52)
Alcohol (YES)	-0.051	0.146	-0.351	0.726	0.95 (0.71,1.27)
Education(High school or equivalent)	0.225	0.112	2	0.045	1.25 (1.01,1.56)
Education(Less than high school)	0.297	0.130	2.28	0.023	1.345 (1.04,1.74)
Personal history of diabetes(YES)	0.040	0.135	0.295	0.768	1.041 (0.80,1.36)
Marital status(Separated/Never/refused)	0.018	0.105	0.169	0.865	1.018 (0.83,1.25)
Marital status(Widowed/Divorced)	0.134	0.116	1.16	0.248	1.14( 0.91,1.43)
PIR(≥1)	-0.461	0.094	-4.92	0	0.631 (0.523,0.76)
WQS: weighted quantile sum; OR:odds ratio; CI:confidence interval.

## Appendix

**Appendix Table A1** (https://www.quintessence-publishing.com/quintessenz/journals/articles/downloads/ohpd_2025_7982_he_suppl_tables.pdf)

**Table A4:** I Information of exposure and outcome data sources.

Consortium	Exposure/Outcome	Cases	Controls	Sample size	Population (Year)	PubMed ID/GWAS ID
GWAS catalog	dental caries	2906	453442	456348	European (2021)	“https://www.ebi.ac.uk/gwas/publications/34737426” “https://www.ebi.ac.uk/gwas/publications/34737426”
GWAS catalog	vitamin A	NA	4959	European(2023)	3727765
GWAS catalog	vitamin B1	NA	4180	European(2023)	“https://www.ebi.ac.uk/gwas/publications/37277652” “https://www.ebi.ac.uk/gwas/publications/37277652”
IEU OpenGWAS	vitamin B2	NA	3301	European(2018)	29875488
GWAS catalog	vitamin B6	NA	1836	European(2023)	“https://www.ebi.ac.uk/gwas/publications/37277652” “https://www.ebi.ac.uk/gwas/publications/37277652”
IEU OpenGWAS	vitamin B12	1707	211115	212822	European(2021)	finn-b-D3_ANAEMIA_B12_DEF
GWAS catalog	vitamin C	NA	2955	European(2021)	“https://www.ebi.ac.uk/gwas/publications/37277652” “https://www.ebi.ac.uk/gwas/publications/37277652”
IEU OpenGWAS	vitamin K	NA	10708	European(2020)	33328453
GWAS catalog	vitamin E	NA	64979	European(2018)	ukb-b-6888


**Table A3:** Inflammatory markers mediating the association between vitamin B12,vitamin E and dental caries.

Exposure	Mediation	ACME	ADE	Total effect	Proportion mediated
Estimate	95%CI lower	95%CI upper	P-value	Estimate	95%CI lower	95%CI upper	P-value	Estimate	95%CI lower	95%CI upper	P-value	Estimate	95%CI lower	95%CI upper	P-value
vitamin B12	CRP	-8.49E-05	-2.16E-04	0	0.036	-9.25E-03	-1.59E-02	0	0.002	-2.52E-02	-1.59E-02	0	0.002	9.05E-03	5.71E-04	0.03	0.038
vitamin B12	leukocyte	9.22E-05	1.59E-06	0	0.046	-9.66E-03	-1.62E-02	0	<2e-16	-9.57E-03	-1.61E-02	0	<2e-16	-9.58E-03	-2.61E-02	0	0.046
vitamin B12	neutrophil	1.10E-04	1.54E-05	0	0.008	-9.57E-03	-1.67E-02	0	<2e-16	-9.46E-03	-1.67E-02	0	<2e-16	-1.16E-02	-3.04E-02	0	0.008
vitamin B12	lymphocyte	-9.57E-05	-2.24E-04	0	0.024	-9.24E-03	-1.63E-02	0	<2e-16	-9.33E-03	-1.64E-02	0	<2e-16	1.02E-02	1.41E-03	0.03	0.024
vitamin E	CRP	-1.72E-04	-4.21E-04	0	0.072	-0.012662	-0.016623	-0.01	<2e-16	-1.28E-02	-1.68E-02	-0.01	<2e-16	1.33E-02	-8.35E-04	0.03	0.072
vitamin E	leukocyte	-3.04E-05	-1.58E-04	0	0.6	-0.0129	-0.0173	-0.01	<2e-16	-1.29E-02	-1.73E-02	-0.01	<2e-16	2.35E-03	-6.12E-03	0.01	0.6
vitamin E	neutrophil	7.85E-06	-7.94E-05	0	0.79	-0.0131	-0.0167	-0.01	<2e-16	-1.31E-02	-1.67E-02	-0.01	<2e-16	-5.98E-04	-8.54E-03	0.01	0.79
vitamin E	lymphocyte	-1.03E-04	-2.39E-04	0	0.032	-0.012953	-0.016594	-0.01	<2e-16	-0.013055	-0.016653	-0.01	<2e-16	7.88E-03	5.13E-04	0.02	0.032
Abbreviations:CRP,C-reactive protein.SNN,Segmented neutrophils num.ACME,average causal mediation effect,ADE,average direct effect.

**Table A2:** PIPs of single vitamins for the prevalence of dental caries in BKMR model.

Variable	PIP
vitamin A	0.292
vitamin B1	0.186
vitamin B2	0.136
vitamin B6	0.894
vitamin B12	0.998
vitamin C	0.624
vitamin K	0.338
vitamin E	0.996
This model adjusted for all covariates and vitamins.PIP, posterior inclusion probability;BKMR,Bayesian kernel machine regression;

**Table A5:** Genetic variants used as instruments in the two-sample Mendelian randomization analyses.

Exosure	SNP	Effect allele	Other allele	EAF	Beta	SE	p_value	F-statistic
vitamin A	rs4436435	A	G	0.270	0.044	0.010	4.01E-06	21.253
vitamin A	rs11608	A	G	0.710	0.051	0.009	5.76E-08	29.430
vitamin A	rs3860447	A	G	0.870	0.062	0.013	1.47E-06	23.179
vitamin A	rs60975479	G	A	0.090	0.077	0.015	1.65E-07	27.397
vitamin A	rs12505657	G	T	0.250	0.046	0.010	3.68E-06	21.418
vitamin A	rs32036	A	T	0.600	0.041	0.009	2.74E-06	21.981
vitamin A	rs10500040	G	A	0.120	0.061	0.013	4.41E-06	21.070
vitamin A	rs10280945	T	G	0.310	-0.045	0.009	1.36E-06	23.324
vitamin A	rs2126259	C	T	0.910	0.085	0.015	1.24E-08	32.404
vitamin A	rs190331487	A	G	0.010	-0.173	0.038	4.47E-06	21.042
vitamin A	rs2806518	G	A	0.610	0.040	0.009	4.98E-06	20.839
vitamin A	rs138492042	A	G	0.020	-0.205	0.039	1.84E-07	27.187
vitamin A	rs8052390	G	A	0.030	-0.118	0.025	2.68E-06	22.025
vitamin B1	rs12082200	T	C	0.180	0.218	0.046	2.29E-06	22.320
vitamin B1	rs4525734	C	G	0.510	-0.164	0.036	3.64E-06	21.438
vitamin B1	rs4954491	T	C	0.280	0.232	0.040	5.33E-09	34.049
vitamin B1	rs151096535	T	C	0.010	0.898	0.185	1.21E-06	23.544
vitamin B1	rs3851364	C	T	0.770	-0.197	0.043	4.10E-06	21.206
vitamin B1	rs1011972	A	T	0.150	-0.234	0.050	2.97E-06	21.824
vitamin B1	rs118174540	T	C	0.010	0.821	0.161	3.40E-07	25.998
vitamin B1	rs7126709	T	C	0.340	-0.176	0.038	2.73E-06	21.988
vitamin B1	rs113567015	G	A	0.020	0.751	0.164	4.43E-06	21.057
vitamin B1	rs2274554	A	G	0.390	0.188	0.037	3.82E-07	25.772
vitamin B1	rs721910	A	C	0.370	0.182	0.037	9.65E-07	23.985
vitamin B1	rs144464382	G	C	0.010	0.739	0.161	4.64E-06	20.969
vitamin B1	rs71371899	A	G	0.010	0.823	0.180	4.75E-06	20.924
vitamin B1	rs117202318	C	T	0.030	-0.594	0.123	1.50E-06	23.135
vitamin B1	rs138519178	G	C	0.030	0.535	0.116	3.66E-06	21.426
vitamin B1	rs145662402	A	T	0.040	0.476	0.101	2.66E-06	22.035
vitamin B1	rs4818790	C	T	0.580	-0.172	0.037	3.94E-06	21.282
vitamin B2	rs9455025	A	G	0.137	-0.163	0.036	4.68E-06	20.885
vitamin B2	rs6930223	T	G	0.499	-0.115	0.024	2.40E-06	22.239
vitamin B2	rs9345080	T	A	0.019	0.459	0.096	1.62E-06	23.020
vitamin B2	rs6993770	T	A	0.282	0.140	0.027	3.47E-07	25.979
vitamin B2	rs117540226	G	C	0.020	-0.433	0.090	1.41E-06	23.268
vitamin B2	rs7182560	T	C	0.522	0.120	0.025	2.29E-06	22.381
vitamin B2	rs74725931	C	T	0.044	0.305	0.065	2.82E-06	21.913
vitamin B2	rs62143194	G	C	0.224	0.389	0.030	1.70E-38	168.119
vitamin B6	rs146502613	T	C	0.030	0.681	0.137	7.37E-07	24.490
vitamin B6	rs12022830	G	A	0.090	0.347	0.073	2.05E-06	22.520
vitamin B6	rs36038454	A	G	0.040	0.503	0.108	2.91E-06	21.852
vitamin B6	rs184343528	A	G	0.010	1.011	0.195	2.28E-07	26.752
vitamin B6	rs35330898	A	G	0.160	-0.268	0.057	2.93E-06	21.837
vitamin B6	rs111918424	G	A	0.010	0.865	0.189	4.58E-06	20.981
vitamin B6	rs80312299	A	C	0.020	0.827	0.175	2.18E-06	22.402
vitamin B6	rs78547201	G	C	0.010	1.154	0.213	6.26E-08	29.248
vitamin B6	rs115266838	T	C	0.010	0.933	0.197	2.19E-06	22.394
vitamin B6	rs79410906	C	T	0.050	0.488	0.102	1.87E-06	22.698
vitamin B6	rs72660033	A	G	0.030	0.632	0.131	1.41E-06	23.238
vitamin B6	rs112902027	G	A	0.010	1.012	0.213	1.97E-06	22.595
vitamin B6	rs144158929	A	T	0.010	0.944	0.206	4.70E-06	20.934
vitamin B6	rs117639748	T	C	0.010	1.238	0.257	1.50E-06	23.126
vitamin B6	rs4471427	G	A	0.970	-0.603	0.121	5.67E-07	24.993
vitamin B6	rs11841410	C	A	0.170	0.260	0.057	4.33E-06	21.092
vitamin B6	rs72713132	T	C	0.010	0.911	0.186	9.14E-07	24.074
vitamin B6	rs142154566	A	T	0.010	1.197	0.241	6.59E-07	24.705
vitamin B12	rs6679677	A	C	0.147	0.519	0.052	2.54E-23	98.853
vitamin B12	rs1990760	T	C	0.585	0.209	0.036	5.09E-09	34.180
vitamin B12	rs17292123	G	A	0.061	0.361	0.075	1.50E-06	23.166
vitamin B12	rs17656368	T	C	0.562	-0.163	0.036	4.56E-06	21.041
vitamin B12	rs7634746	G	C	0.327	0.180	0.038	1.90E-06	22.701
vitamin B12	rs35896106	T	C	0.080	0.303	0.066	4.69E-06	20.957
vitamin B12	rs9321367	T	G	0.128	-0.255	0.054	2.02E-06	22.598
vitamin B12	rs28407950	T	C	0.344	-0.286	0.042	1.16E-11	46.027
vitamin B12	rs9270535	A	G	0.425	-0.220	0.040	2.42E-08	31.077
vitamin B12	rs140650994	C	G	0.001	2.510	0.547	4.50E-06	21.039
vitamin B12	rs79132259	T	G	0.033	0.487	0.102	1.99E-06	22.581
vitamin B12	rs75973258	G	A	0.013	0.767	0.168	4.75E-06	20.929
vitamin B12	rs7310615	G	C	0.586	-0.171	0.036	1.71E-06	22.943
vitamin B12	rs151234	C	G	0.124	0.305	0.054	2.05E-08	31.413
vitamin B12	rs35056955	G	A	0.008	1.031	0.222	3.28E-06	21.644
vitamin B12	rs73597298	G	T	0.110	-0.309	0.058	8.58E-08	28.642
vitamin B12	rs2826921	A	C	0.221	0.217	0.043	3.32E-07	26.043
vitamin C	rs558596774	C	G	0.020	-1.598	0.326	9.73E-07	23.965
vitamin C	rs10493976	T	C	0.170	0.485	0.101	1.50E-06	23.129
vitamin C	rs4853943	T	C	0.110	-0.627	0.125	5.77E-07	24.972
vitamin C	rs3979146	A	T	0.810	0.475	0.098	1.38E-06	23.296
vitamin C	rs41334350	A	G	0.090	-0.677	0.139	1.17E-06	23.606
vitamin C	rs10275348	C	T	0.370	-0.384	0.080	1.85E-06	22.724
vitamin C	rs7037929	A	C	0.430	0.364	0.079	4.07E-06	21.215
vitamin C	rs12761049	T	A	0.100	0.595	0.130	4.81E-06	20.899
vitamin C	rs12830213	A	C	0.370	-0.379	0.080	2.25E-06	22.351
vitamin K	rs72669092	A	G	0.020	0.237	0.052	4.35E-06	21.120
vitamin K	rs60229162	A	G	0.012	-0.372	0.064	5.56E-09	34.009
vitamin K	rs2493137	C	T	0.298	-0.069	0.015	2.40E-06	22.202
vitamin K	rs1799810	T	A	0.427	-0.157	0.013	7.69E-32	137.949
vitamin K	rs113526757	C	T	0.013	0.317	0.061	1.67E-07	27.414
vitamin K	rs1728918	G	A	0.717	-0.079	0.015	1.17E-07	28.014
vitamin K	rs11720167	G	T	0.206	-0.097	0.017	2.00E-08	31.497
vitamin K	rs11753042	A	G	0.076	0.131	0.026	4.66E-07	25.422
vitamin K	rs6913296	G	A	0.051	-0.143	0.031	3.53E-06	21.443
vitamin K	rs149350586	A	G	0.013	-0.418	0.090	3.72E-06	21.397
vitamin K	rs6479298	G	A	0.671	-0.069	0.014	1.22E-06	23.549
vitamin K	rs2458675	A	C	0.255	0.074	0.016	1.76E-06	22.727
vitamin K	rs17433712	T	C	0.034	0.185	0.038	8.61E-07	24.204
vitamin K	rs80032902	C	T	0.036	-0.176	0.036	1.16E-06	23.633
vitamin K	rs146039650	T	C	0.016	0.268	0.055	1.07E-06	23.823
vitamin K	rs743271	A	G	0.674	-0.069	0.015	3.12E-06	21.795
vitamin K	rs7187954	C	G	0.129	0.092	0.020	4.54E-06	21.037
vitamin K	rs55864960	A	G	0.026	-0.208	0.045	4.19E-06	21.140
vitamin K	rs35489850	C	T	0.209	-0.079	0.017	2.93E-06	21.755
vitamin K	rs11904888	A	T	0.093	0.556	0.022	2.55E-138	627.814
vitamin K	rs6088232	T	C	0.018	0.338	0.054	2.64E-10	39.852
vitamin K	rs113645189	C	T	0.039	-0.194	0.036	7.67E-08	28.934
vitamin K	rs77621298	G	A	0.135	0.094	0.021	4.59E-06	20.932
vitamin E	rs536912	A	C	0.736	0.030	0.006	9.00E-07	24.128
vitamin E	rs6033	G	A	0.072	-0.052	0.011	9.90E-07	23.945
vitamin E	rs2723979	G	T	0.584	-0.027	0.006	1.50E-06	23.183
vitamin E	rs979218	C	A	0.098	-0.043	0.009	3.10E-06	21.771
vitamin E	rs79966958	T	C	0.013	-0.117	0.025	2.00E-06	22.612
vitamin E	rs12421920	G	A	0.094	-0.043	0.009	3.70E-06	21.432
vitamin E	rs111306778	A	G	0.090	-0.048	0.010	5.40E-07	25.108
vitamin E	rs4903544	T	C	0.300	-0.030	0.006	9.50E-07	24.020
vitamin E	rs12899673	A	G	0.333	0.027	0.006	3.80E-06	21.369
vitamin E	rs35218694	G	A	0.034	-0.074	0.015	1.30E-06	23.464
vitamin E	rs71385328	G	A	0.011	0.130	0.026	7.00E-07	24.613
vitamin E	rs12165526	A	T	0.101	0.048	0.009	1.80E-07	27.241


**Table A6:** The MR analysis results of 5 methods (Inverse variance weighted, MR Egger, Maximum likelihood, Weighted median, Penalised weighted median).

Exposure	p_value	OR	OR_lci95	OR_uci95	Heterogeneity P-value	Pleiotropy P-value	MR-PRESSO global test RSSobs value
Cochran’s Q test	MR-Egger intercept test	
vitamin A					0.679	0.632	
Inverse variance weighted	0.829	1.042	0.715	1.520			
MR Egger	0.759	0.850	0.310	2.329			
Maximum likelihood	0.823	1.045	0.713	1.531			
Simple median	0.857	1.052	0.606	1.826			
Weighted median	0.831	1.057	0.635	1.758			
Penalised weighted median	0.832	1.057	0.633	1.763			
vitamin B1					0.264	0.925	
Inverse variance weighted	0.895	0.995	0.919	1.076			
MR Egger	0.362	1.079	0.922	1.262			
Maximum likelihood	0.896	0.995	0.919	1.077			
Simple median	0.913	0.994	0.900	1.099			
Weighted median	0.852	0.990	0.894	1.097			
Penalised weighted median	0.854	0.990	0.893	1.098			
vitamin B2					0.263	0.605	
Inverse variance weighted	0.305	1.064	0.945	1.198			
MR Egger	0.164	1.210	0.956	1.532			
Maximum likelihood	0.301	1.065	0.945	1.201			
Simple median	0.330	1.091	0.916	1.299			
Weighted median	0.206	1.098	0.950	1.269			
Penalised weighted median	0.207	1.098	0.950	1.269			
vitamin B6					0.838	0.995	
Inverse variance weighted	0.412	0.976	0.920	1.035			
MR Egger	0.569	0.965	0.856	1.088			
Maximum likelihood	0.408	0.975	0.920	1.035			
Simple median	0.655	0.982	0.907	1.063			
Weighted median	0.606	0.981	0.910	1.056			
Penalised weighted median	0.624	0.981	0.907	1.060			
vitamin B12					0.320	0.883	0.907
Inverse variance weighted	0.021	1.061	1.009	1.115			
MR Egger	0.457	1.030	0.955	1.110			
Maximum likelihood	0.024	1.062	1.008	1.119			
Simple median	0.216	1.049	0.972	1.132			
Weighted median	0.219	1.048	0.972	1.130			
Penalised weighted median	0.218	1.048	0.972	1.130			
vitamin C					0.612	0.558	
Inverse variance weighted	0.943	0.998	0.950	1.048			
MR Egger	0.610	0.941	0.755	1.174			
Maximum likelihood	0.942	0.998	0.950	1.049			
Simple median	0.679	0.988	0.930	1.048			
Weighted median	0.731	0.989	0.928	1.054			
Penalised weighted median	0.737	0.989	0.927	1.055			
vitamin K					0.510	0.617	
Inverse variance weighted	0.085	0.901	0.800	1.015			
MR Egger	0.515	0.942	0.790	1.123			
Maximum likelihood	0.091	0.902	0.800	1.016			
Simple median	0.269	0.879	0.699	1.105			
Weighted median	0.751	0.976	0.841	1.133			
Penalised weighted median	0.752	0.976	0.841	1.134			
vitamin E					0.522	0.419	
Inverse variance weighted	0.904	0.964	0.533	1.746			
MR Egger	0.584	1.569	0.331	7.435			
Maximum likelihood	0.904	0.964	0.530	1.753			
Simple median	0.893	0.943	0.404	2.206			
Weighted median	0.897	0.947	0.417	2.150			
Penalised weighted median	0.900	0.947	0.408	2.202			

